# Vitamin D binding protein correlate with estrogen increase after administration of human chorionic gonadotropin but do not affect ovulation, embryo, or pregnancy outcomes

**DOI:** 10.3389/fendo.2024.1401975

**Published:** 2024-05-23

**Authors:** Huijun Chen, Jianghui Yao, Liang Hu, Yvonne Liu, Johann-Georg Hocher, Xiaoli Zhang, Ahmed A. Hasan, Ge Lin, Fei Gong, Berthold Hocher

**Affiliations:** ^1^School of Basic Medicine, Central South University, Changsha, Hunan, China; ^2^Clinical Research Center for Reproduction and Genetics in Hunan Province, Reproductive and Genetic Hospital of CITIC-Xiangya, Changsha, Hunan, China; ^3^Department of Nephrology, Charité Universitätsmedizin Berlin, Berlin, Germany; ^4^Key Laboratory of Stem Cells and Reproductive Engineering, Ministry of Health, Changsha, China; ^5^Fifth Department of Medicine (Nephrology/Endocrinology/Rheumatology/Pneumology), University Medical Centre Mannheim, University of Heidelberg, Mannheim, Germany; ^6^Institute of Medical Diagnostics (IMD), Berlin, Germany

**Keywords:** vitamin D binding protein, 25(OH)D, oocyte quality, embryo quality, pregnancy outcomes

## Abstract

**Background:**

Vitamin D binding protein (DBP) might increase substantially after ovarian stimulation and hence could be associated with IVF/ICSI outcomes because it determines the fraction of free bioavailable 25(OH) vitamin D. In this study, we aim to determine whether DBP is associated with E2 level after ovarian stimulation and IVF/ICSI outcomes.

**Design:**

*Post-hoc* analysis of a prospective observational cohort.

**Setting:**

Single-center study.

**Participants:**

2569 women receiving embryo transfer.

**Intervention:**

None.

**Main outcome measures:**

The main outcomes were oocyte and embryo quality as well as pregnancy outcomes.

**Results:**

DBP concentration correlates with E2 on hCG day (=day of inducing ovulation with hCG; correlation coefficient r = 0.118, P<0.001) and E2 x-fold change to baseline level (r = 0.108, P<0.001). DBP is also positively correlated with total 25(OH)D (r = 0.689, R^2^ = 0.475, P<0.001) and inversely with free 25(OH)D (r=-0.424, R^2^=0.179, P<0.001), meaning that E2-stimulated DBP synthesis results in a decrease of free 25(OH)D during ovarian stimulation. However, such alteration does not affect IVF/ICSI outcomes when considering confounding factors, such as the number and quality of oocytes nor embryo quality as well as pregnancy outcomes.

**Conclusion:**

DBP concentration correlates with the degree of E2 increase after ovarian stimulation. DBP is also positively correlated with total 25(OH)D and inversely with free 25(OH)D, suggesting that the proportion of free 25(OH)D decreases during ovarian stimulation caused by E2-stimulated DBP synthesis. However, such alteration does not affect clinical IVF/ICSI outcomes.

## Introduction

Vitamin D is a fat-soluble steroid (1). After its synthesis, vitamin D is transformed to 25-hydroxyvitamin D (25(OH)D) by 25-hydroxylase in the liver. Over 99% of 25(OH)D is bound to either vitamin D-binding protein (DBP) (about 85%) or albumin (about 15%), leaving less than 1% free, which can pass through the lipophilic cell membrane and interact directly or after further hydroxylation with the nuclear vitamin D receptor (2, 3).

DBP stores and transports vitamin D and regulates the amounts of circulating free and total levels of vitamin D metabolites (4, 5). It is a protein with 458 amino acids, synthesized in the liver, where it is regulated by estrogen, glucocorticoids, and inflammatory cytokines but not by vitamin D itself (6). An interesting study published in the New England Journal of Medicine showed that total 25(OH)D concentrations were consistently lower in black Americans compared to white Americans. However, they had similar concentrations of estimated free, bioavailable 25(OH)D, due to different levels of DBP (7). This study demonstrated the importance of DBP when analyzing vitamin D levels, as DBP concentration and variants alter the proportion of 25(OH)D metabolites. A previous study in mice lacking DBP showed very low levels of total 25(OH)D but the animals did not show signs of vitamin D deficiency (8). Thus, DBP serves as a reservoir for vitamin D metabolites, reducing the risk of vitamin D deficiency when intake or epidermal production is limited (4). Recent clinical studies tried to further understand the impacts of DBP in different clinical settings. Findings and hence clinical implications were, however, controversial. For example, high circulating DBP concentrations were found to be associated with better mobility and reduced mortality after hip fracture surgery (9). On the other hand, colorectal cancer mortality did not differ according to DBP variants in two US cancer cohorts (10). In another study, mortality risks were similar across DBP quintiles in aging men, whereas 25(OH)D deficiency was associated with a 2-fold increased mortality (11).

There are also studies focusing on DBP and reproductive health. These studies showed that DBP concentrations are lower in women with polycystic ovary syndrome (PCOS) compared to controls (median [interquartile range]: 443.40 [314.4] vs 482.4 [156.8] μg/ml, P=0.02) (12, 13) and that urinary DBP is associated with ovarian reserve (normal control 81.86 ± 23.92 and diminished ovarian reserve 52.84 ± 21.37 ng/ml, P< 0.05) and it was significantly greater in patients with endometriosis than in those without (111.96 ± 74.59 versus 69.90 ± 43.76 ng/mg Cr, P = 0.001) (14, 15). DBP has been shown to increase by 40–50% during pregnancy due to a physiological increase in estrogen (E2) (16). Interestingly, Hou et al. state their results that DBP is highly expressed in the placenta and the decidua in women with spontaneous miscarriages, potentially causing a less efficient transport of vitamin D to the fetus (17). Further studies showed that DBP is related to pregnancy complications such as pre-eclampsia (up-regulated with fold changes 3.38 in early-onset preeclampsia) (18, 19), gestational diabetes (lower DBP concentrations were associated with higher glucose levels and a greater likelihood of developing GDM at 26–28 weeks gestation (odds ratio [OR] (95% CI) = 0.98 (0.97,0.99), P = 0.015)) (20), preterm birth (DBP is reduced, P=0.04) (21, 22), fetal growth restriction (DBP was significantly reduced (control versus FGR, P< 0.05) and strongly associated with idiopathic fetal growth restriction (P< 0.01)) (23) as well as reduced birthweight (24).

Given these findings and the fact that E2, which stimulates DBP synthesis in the liver, rises substantially after ovarian stimulation in women undergoing *in-vitro* fertilization/intracytoplasmic sperm injection (IVF/ICSI), DBP might also increase substantially and hence could be associated with IVF/ICSI outcomes. However, there is no published study focusing on the serum concentration of DBP and its impact on IVF outcomes in animals or humans so far.

The purpose of our current study is to analyze the impact of ovarian stimulation in women undergoing IVF/ICSI on DBP and its potential consequences for clinical IVF/ICSI outcomes.

## Materials and methods

### Study design

This is a *post-hoc* analysis of our previous cohort data (25), which were collected from January 2017 to December 2018. The initial study was registered at **Clinical.trial.gov, the number is:** NCT03503006 (on clinicaltrials.gov) https://clinicaltrials.gov/study/NCT03503006?locStr=Changsha,%20Hunan,%20China&country=China&state=Hunan&city=Changsha&cond=ivf&rank=2.

The study was approved by the Ethics Committee of the Reproductive and Genetic Hospital of CITIC-Xiangya (approval number: LL-SC-2018–014) and written consent was obtained from all participating patients.

### Participants

A total of 2569 infertile patients were enrolled in this study. The inclusion and exclusion criteria were clearly described in the previous study (25, 26), which were as follows:

Inclusion criteria:

[1] age between 18 and 40 years old
[2] first IVF/ICSI cycle
[3] received fresh embryo transfer.

The exclusion criteria were:

[1] uterine malformations (uterine septum ≥0.6 cm (identified by hysteroscopy or four-dimensional color Doppler ultrasound), single- or double-horned uterus)
[2] endometriosis
[3] Asherman syndrome (intrauterine adhesion)
[4] untreated hydrosalpinx
[5] uterine myoma (multiple, submucous, or intramural myoma >3 cm)
[6] oocyte donation cycles
[7] pre-implantation genetic test for aneuploidy (PGT-A)
[8] Cushing syndrome
[9] adult-onset adrenogenital syndrome (AGS)
[10] any hypothalamic or pituitary disease leading to infertility.

These in-and-exclusion criteria were chosen to study a population where clearly defined reasons for infertility such as uterine malformations or adenoma of the hypophysis that are independent of the vitamin D system do not play a role.

All participants received an agonist protocol for ovarian stimulation as described in our previous studies (25, 26). Total and free vitamin D were measured after ovarian stimulation, one day before embryo transfer according to the manufacturer’s instructions (25).

### Measuring albumin, total and free vitamin D

All serum samples were collected one day before embryo transfer in women and were kept frozen at −80°C until measurement. Total and free 25(OH)D were measured using ELISA (DIAsource ImmunoAssays, Belgium) as detailed described in our previous work (25, 27, 28). Initially, we chose the necessary number of strips for the experiment, resealing any unused strips in the bag with a desiccant and storing them at 2 – 8°C. Subsequently, the strips were secured in the holding frame, and 90 µl of sample diluent was pipetted into all the wells. Following that, 10 µl of each reconstituted calibrator, control, or sample was pipetted in duplicate into the appropriate wells, employing a new pipette tip for each sample. The assay plate underwent a 90-minute incubation at 37°C, with shaking at 650 rpm, and was then subjected to three washes with 350 µl wash buffer. In the next step, 100 µl of working Biot-Vit D solution was added to all wells and incubated for 30 minutes at 37°C, with shaking at 650 rpm. The plate was washed three times with 350 µl wash buffer. Subsequently, 100 µl of Streptavidin-HRP reagent was introduced into all wells, followed by a 20-minute incubation at 37°C, with shaking at 650 rpm. Another three washes with 350 µl wash buffer were performed. Next, 100 µl of the chromogenic solution was added to all wells and incubated for 15 minutes at room temperature (18–25°C), in a stationary position and shielded from light. Finally, 100 µl of the stop reagent was added to all wells, and absorbances at 450 nm were read within 5 minutes (using a reference filter at 630nm or 650nm), with results calculated according to the guidelines in Section XI. Albumin was measured by colorimetry according to the manufacturer’s instructions (Albumin Gen 2(ALB2), Roche Diagnostics GmbH, Mannheim, Germany).

### Vitamin D binding protein calculation

Free vitamin D can be detected either directly or indirectly using the following formula (2, 29):


$$Free\;25\;\left(OH\right)D=\frac{Total\;25\;\left(OH\right)D}{1+Kalb\times albumin+KDBP\times DBP}$$

Kalb is the affinity constant between 25(OH)D and albumin, which equals $6\times 10^{5}\;M^{-1}$ . KDBP is the affinity constant between 25(OH)D and DBP, which equals $7\times 10^{8}M^{-1}$ according to Bickle’s. study (29).

In our study, we measured free vitamin D directly and transformed the formula to calculate DBP level. This was possible since albumin level was also measured directly. The transformed formula was as follows:


$$DBP=\frac{\frac{Total\;25\left(OH\right)D}{Free\;25\left(OH\right)D}-1-Kalb\times albumin}{KDBP}$$

### Statistical analysis

Statistical Package for Social Sciences for Windows, version 29.0 (SPSS Inc, Chicago, IL, USA) was used to perform data analyses. The homogeneity of variance and normality of data were estimated using the Levene and Kolmogorov-Smirnov tests, respectively. Values were expressed as medians (interquartile ranges), means (± standard deviation), or frequency (%). A comparison of quantitative variables between groups was performed using the Kruskal-Wallis test or ANOVA according to the data’s normality. Qualitative variables were compared with the Chi-square test or Fisher’s exact test. Pearson and Spearman correlation analysis was performed for the correlation analysis according to the data category. To adjust for potential confounders, we performed multivariate linear regression analyses. These confounding factors were determined by comparing the baseline characteristics. Figures were created in GraphPad Prism 8 (GraphPad Software, San Diego, USA). Data were considered statistically significant with a two-sided P< 0.05.

## Results

**Figure 1:**
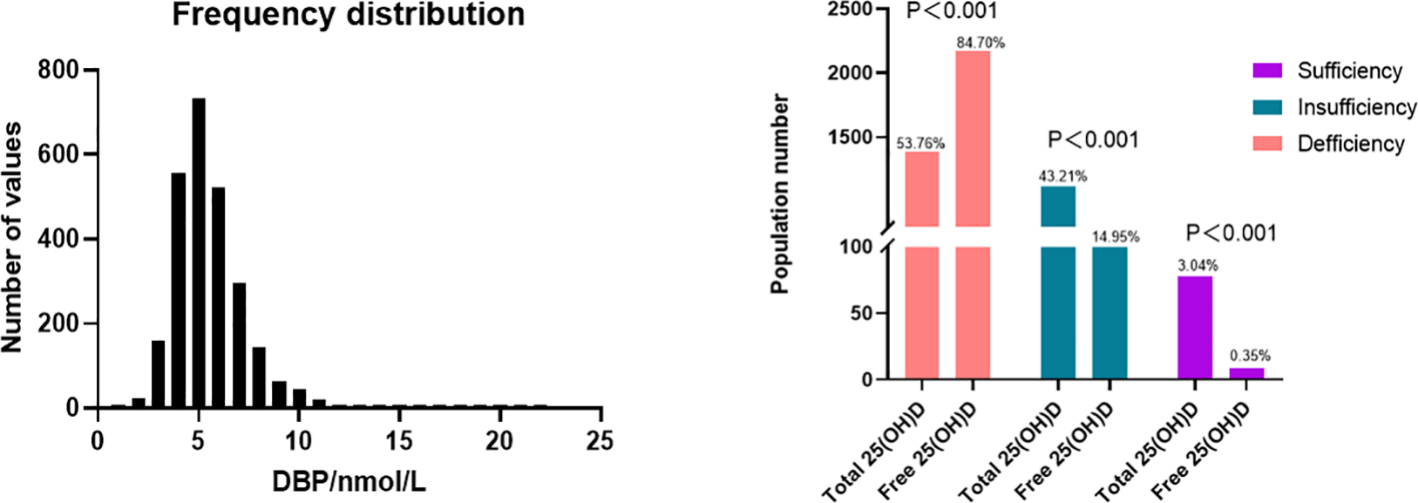
Distribution of DBP and 25(OH)D level. Top: The distribution of DBP (n = 2549), mean = 5.494 nmol/L, 25% percentile = 4.259nmol/L, 75% percentile = 6.356 nmol/L. According to the current guideline, total 25(OH)D sufficiency is determined as at least 30 ng/mL (75 nmol/L) while insufficiency and deficiency are defined as 20-<30 ng/mL (50–75nmol/L), and below 20 ng/mL (50nmol/L) respectively (30, 31). Zeng et al. determined corresponding cut-off values for free 25(OH)D, where free 25(OH)D sufficiency was defined as over 8.499 pg/mL, while insufficiency and deficiency are defined as 5.666–8.499 pg/mL and below 5.666 pg/mL respectively (32).

A total of 2569 participants fulfilling all in- and exclusion criteria were analyzed. The DBP concentration distribution is shown in **Figure 1**. Baseline clinical and laboratory parameters were shown according to DBP quartiles (**Table 1**). Age, menstrual cycle length, anti-Müllerian hormone (AMH), antral follicle count (AFC), albumin as well as free and total 25(OH)D were significantly different in the DBP quartiles.

**Table 1:** Demographic data of participants according to quartiles of vitamin D binding protein (DBP).

	Q1 (n=642)	Q2 (n=642)	Q3 (n=642)	Q4 (n=643)	P value
Age (y)	29.00 (27.00,31.00)	29.00 (27.00,31.00)	29.00 (27.00,31.25)	29.00 (27.00,32.00)	0.041
Menstrual cycle (d)	30.00 (28.50, 34.00)	30.00 (29.00, 35.00)	30.50 (29.00, 37.50)	30.50 (29.00, 37.50)	<0.001
Infertility type (%)					
Primary	55.61 (357/642)	54.36 (349/642)	51.87 (333/642)	50.86 (327/643)	0.294
Secondary	44.39 (285/642)	45.64 (293/642)	48.13 (309/642)	49.14 (316/643)	
BMI (kg/m2)	21.23 (19.72, 23.05)	21.44 (19.77, 23.11)	21.36 (19.72, 23.11)	21.30 (19.49, 22.94)	0.451
Waist-to-hip ratio	0.81 (0.78, 0.85)	0.81 (0.78, 0.85)	0.82 (0.78, 0.85)	0.81 (0.78, 0.85)	0.842
Infertility duration (y)	3.00 (2.00, 4.00)	3.00 (2.00, 5.00)	3.00 (2.00, 5.00)	3.00 (2.00, 5.00)	0.203
AMH (ng/ml)	5.25 (3.37, 8.24)	5.60 (3.52, 8.90)	5.93 (3.82, 10.07)	6.24 (3.90, 10.05)	<0.001
AFC	21.00 (15.00, 30.00)	23.00 (16.00, 30.00)	25.00 (17.00, 30.00)	25.00 (17.00, 30.00)	<0.001
Basal FSH (mIU/ml)	5.70 (4.91, 6.62)	5.59 (4.74, 6.60)	5.60 (4.72, 6.50)	5.59 (4.78, 6.40)	0.141
Basal LH (mIU/ml)	3.58 (2.64, 4.90)	3.58 (2.59, 4.85)	3.65 (2.69,5.36)	3.57 (2.55, 4.90)	0.267
Basal E2 (pg/ml)	33.00 (27.00, 42.00)	34.00 (27.00, 43.25)	34.00 (27.00, 44.00)	33.00 (26.00, 43.00)	0.603
Basal PRL (ng/ml)	14.76 (11.02, 19.80)	14.86 (11.00, 19.59)	14.70 (11.04, 19.72)	14.88 (11.07, 20.86)	0.917
Basal P (ng/ml)	0.24 (0.17, 0.33)	0.24 (0.18, 0.33)	0.23 (0.17, 0.31)	0.24 (0.18, 0.33)	0.623
Basal T (ng/ml)	0.28 (0.22, 0.36)	0.28 (0.23, 0.35)	0.28 (0.23, 0.38)	0.28 (0.22, 0.35)	0.154
Total 25(OH)D (ng/ml)	15.98 (13.94, 18.22)	18.51 (16.21, 20.50)	20.72 (18.41, 23.28)	24.09 (21.03, 26.96)	<0.001
Free 25(OH)D (pg/ml)	5.18 (4.55, 5.83)	4.91 (4.29, 5.35)	4.68 (4.14, 5.18)	4.16 (3.65, 4.71)	<0.001
Albumin (g/L)	49.50 (48.00, 51.10)	49.20 (47.30, 50.90)	49.10 (47.30, 50.80)	48.80 (47.20, 50.50)	<0.001
DBP (x 10^-6^) (g/L)	3.83 (3.43, 4.12)	4.79 (4.57, 4.99)	5.73 (5.47, 6.01)	7.34 (6.75, 8.30)	<0.001

BMI, body mass index; AMH, anti-Müllerian hormone; AFC, antral follicle count; FSH, follicle-stimulating hormone; LH, luteinizing hormone; E2, estradiol; PRL, prolactin; P, progesterone; T, testosterone; DBP, vitamin D binding protein.

Data are given as medians (interquartile ranges) or numbers (percentages).

Q1: DBP=1.55 x 10^-6^-4.35 x 10^-6^ (mmol/L).

Q2: DBP=4.36 x 10^-6^-5.22 x 10^-6^ (mmol/L).

Q3: DBP=5.23 x 10^-6^-6.35 x 10^-6^ (mmol/L).

Q4: DBP≥6.36 x 10^-6^ (mmol/L).

**Figure 2:**
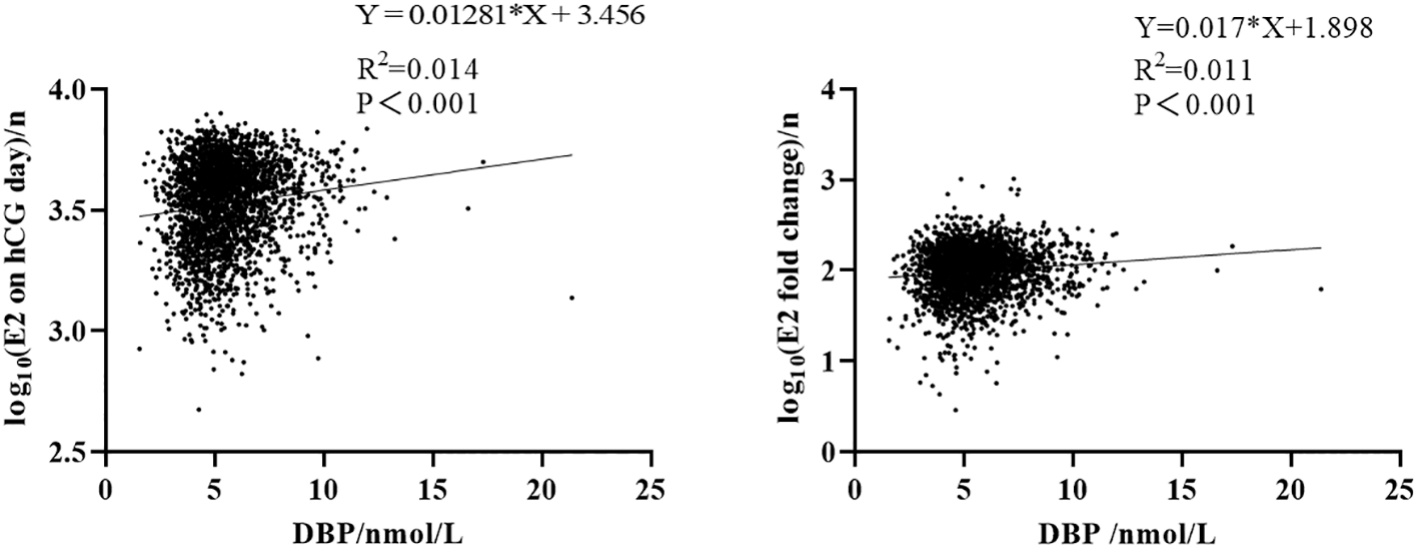
Correlation between DBP and estrogen. Left: correlation between E2 on hCG day and DBP concentration after log_10_ transformation. Right: correlation between E2 x-fold change (baseline E2 level divided by E2 on hCG day) and DBP concentration after log_10_ transformation.

**Figure 3:**
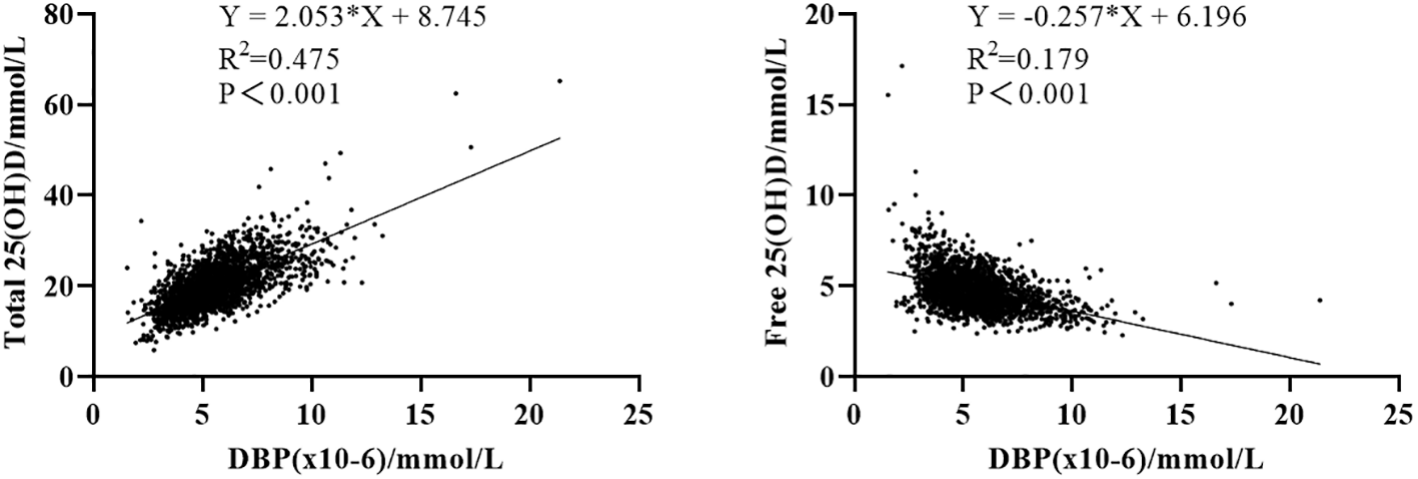
Correlation between 25(OH)D and its binding protein. Left: correlation between total 25(OH)D and DBP concentration. Right: correlation between free 25(OH)D and DBP concentration.

Ovarian stimulation in women undergoing IVF causes a supraphysiological elevation of E2, sometimes increasing several hundred-fold (see the E2 level in **Table 2** compared to **Table 1**). Both the x-fold-change of E2 after ovarian hyperstimulation as well as peak-E2 after ovarian hyperstimulation correlated highly significantly with DBP concentrations after ovarian hyperstimulation on the day before embryo transfer (**Figure 2**). Total 25(OH)D was positively correlated with DBP, whereas free 25(OH)D was inversely correlated with DBP (**Figure 3**). This means that the increased DBP concentration resulted in a decreased fraction of patients being sufficiently supplied with free vitamin D during embryo transfer (**Figure 1**).

**Table 2:** Ovarian hyperstimulation results in participants according to quartiles of vitamin D binding protein (DBP).

	Q1 (n=642)	Q2 (n=642)	Q3 (n=642)	Q4 (n=643)	P value
E2 on hCG day/[pg/ml]	3295.50 (2237.25, 4301.75)	3640.50 (2653.75, 4622.50)	3602.50 (2656.75, 4586.50)	3890.00 (2859.00, 4811.00)	<0.001
P on hCG day/[ng/ml]	0.59 (0.45, 0.79)	0.59 (0.43, 0.79)	0.58 (0.42, 0.79)	0.59 (0.43, 0.82)	0.505
LH on hCG day/[mIU/ml]	1.48 (1.14, 1.93)	1.57 (1.19, 1.97)	1.57 (1.24, 2.01)	1.60 (1.21, 2.06)	0.019
No. of oocytes retrieved	11.00 (8.00, 15.00)	12.00 (9.00, 16.00)	12.00 (9.00, 15.00)	12.00 (9.00, 15.00)	0.002
No. of MII oocytes	10.00 (7.00, 13.00)	11.00 (8.00, 14.00)	10.00 (8.00, 14.00)	11.00 (8.00, 14.00)	0.001
No. of 2PN zygotes	6.00 (4.00, 9.00)	7.00 (5.00, 9.00)	7.00 (4.00, 9.00)	9.00 (4.00, 9.00)	0.166
Fertilization methods (%)					
IVF	69.16 (444/642)	70.56 (453/642)	71.50 (459/642)	67.34 (433/643)	
ICSI	16.36 (105/642)	15.73 (101/642)	15.73 (101/642)	16.80 (108/643)	0.738
IVF+ICSI	14.49 (93/642)	13.71 (88/642)	12.77 (82/642)	15.86 (102/643)	
Fertilization rate (%)	66.67 (54.55, 81.82)	66.67 (50.00, 80.00)	66.67 (50.00, 80.00)	66.67 (50.00, 81.25)	0.229
The number of day 3 good quality embryo	3.94 ± 3.28	3.88 ± 3.28	4.35 ± 3.38	4.35 ± 3.46	0.013
Day 3 good quality embryo rate (%)	69.94 (2532/3619)	66.58 (2488/3737)	71.42 (2791/3908)	72.03 (2794/3879)	<0.001
Blastocyst formation rate (%)	34.52 (651/1886)	33.31 (784/2354)	36.77 (832/2263)	34.19 (719/2103)	0.088
EM before embryo transfer(mm)	13.50 (12.00, 14.70)	13.50 (12.20, 15.00)	13.15 (11.80, 14.50)	13.10 (11.70, 14.50)	0.002
Mean number of transferred embryos	1.91 ± 0.29	1.90 ± 0.30	1.89 ± 0.32	1.91 ± 0.29	0.591
Good-quality embryo transfer rate (%)	79.04 (969/1226)	80.59 (984/1221)	81.93 (993/1212)	81.31 (996/1225)	0.301
Mild to severe OHSS rate (%)	3.74 (24/642)	5.14 (33/642)	4.83 (31/642)	6.69 (43/643)	0.116

LH, luteinizing hormone; E2, estradiol; Gn, gonadotropin; EM, endometrium; hCG, human chorionic gonadotropin; MII, metaphase II (reflects oocytes quality, only MII oocytes can be fertilized); 2PN, pronucleus; IVF, in vitro fertilization; ICSI, intracytoplasmic sperm injection.

Good quality embryo is defined as D3 embryo ≥7C-II and blastocyst ≥4BB while fair embryo is defined as D3 embryo <7C-II and blastocyst <4BB; OHSS: ovarian hyperstimulation syndrome.

Data are given as medians (interquartile ranges), means ± standard deviation or number (percentage).

Q1: DBP=1.55 x 10^-6^-4.35 x 10^-6^ (mmol/L).

Q2: DBP=4.36 x 10^-6^-5.22 x 10^-6^ (mmol/L).

Q3: DBP=5.23 x 10^-6^-6.35 x 10^-6^ (mmol/L).

Q4: DBP≥6.36 x 10^-6^ (mmol/L).

In **Table 2**, early ovarian hyperstimulation outcome parameters are shown in quartiles of DBP. Luteinizing hormone (LH), E2, the number of oocytes obtained and the number of Metaphase II (MII) oocytes, and the number of day three good quality embryos increased from DBP quartile 1 to DBP quartile 4, whereas endometrial thickness before embryo transfer decreased from DBP quartile 1 to DBP quartile 4. This fits with the overall correlation analysis of IVF outcomes and DBP concentrations shown in **Table 3**. Only the number of oocytes, the number of MII oocytes, and the number of day three good quality embryos were significantly correlated with DBP concentrations one day before embryo transfer. Other IVF outcome parameters such as live birth rate or miscarriage rate were not related in this univariate analysis with DBP. To further investigate the correlation of the significantly associated parameters from **Table 3**, we investigated the influence of confounding factors associated with DBP quartiles such as age, menstrual cycle length, AMH, AFC, and albumin. This analysis revealed that there was no independent association between DBP measured one day before embryo transfer and early IVF outcomes (**Table 4**). Only the number of day three good-quality embryos showed a strong trend toward an association with DBP (p = 0.055).

**Table 3:** Correlation analysis between IVF outcomes and vitamin D binding protein (DBP).

	DBP	
	rho	P value
Number of oocytes retrieved	0.054	0.006
Number of MII oocytes	0.055	0.006
Number of 2PN zygotes	0.032	0.101
Fertilization rate	-0.023	0.252
Number of D3 good-quality embryo	0.058	0.003
OHSS rate	0.001	0.975
Biochemical pregnancy	0.020	0.322
Clinical pregnancy	0.013	0.517
Ectopic pregnancy	0.007	0.721
Miscarriage	0.002	0.919
Ongoing pregnancy	0.017	0.381
Live birth	0.011	0.561
Gestational age	0.004	0.862
Birthweight	-0.005	0.835

Spearman correlation analysis between IVF outcomes and DBP concentrations on the day before embryo transfer.

Colored fields indicate significant correlations.

MII, metaphase II (reflects oocytes quality, only MII oocyte can be fertilized); 2PN, pronucleus; OHSS, ovarian hyperstimulation syndrome.

**Table 4:** Multivariate linear regression analysis.

	Number of oocytes retrieved				Number of MII oocytes				Number of D3 good-quality embryos			
	OR	P value	95% CI of OR		OR	P value	95% CI of OR		OR	P value	95% CI of OR	
Constant		<0.001	11.542	18.872		<0.001	9.160	16.017		<0.001	2.200	7.530
Age	-0.109	<0.001	-0.195	-0.093	-0.071	<0.001	-0.134	-0.039	-0.044	0.030	-0.078	-0.004
Menstrual cycle	-0.134	<0.001	-0.040	-0.021	-0.106	<0.001	-0.031	-0.013	-0.065	0.003	-0.018	-0.004
Albumin	-0.004	0.826	-0.070	0.056	-0.009	0.634	-0.073	0.044	-0.009	0.649	-0.056	0.035
DBP	0.030	0.119	-0.022	0.189	0.027	0.158	-0.028	0.170	0.038	0.055	-0.002	0.152
AMH	0.167	<0.001	0.042	0.085	0.156	<0.001	0.035	0.075	0.088	0.003	0.008	0.039
AFC	0.080	0.003	0.025	0.125	0.072	0.009	0.015	0.109	0.075	0.007	0.014	0.086

Multivariate linear regression analysis of IVF outcomes being correlated with DBP in the univariate analysis (see **Table 3**). We selected significantly different baseline parameters among different DBP quartiles (see **Table 1**) as confounding factors. Free 25(OH)D and total 25(OH) were eluded due to collinearity to DBP. We thus hence finally included age, AFC, AMH and DBP in the final multivariate linear regression analysis.

AMH, anti-Müllerian hormone; AFC, antral follicle count; DBP, vitamin D binding protein.

## Discussion

Vitamin D binding protein concentration correlates very well with the degree of E2 increase after ovarian stimulation for oocyte retrieval. DBP is also positively correlated with total 25(OH)D and inversely with free 25(OH)D, suggesting that the proportion of free, bioavailable 25(OH)D decreases during ovarian stimulation caused by E2-stimulated DBP synthesis. However, such alteration does not affect IVF/ICSI outcomes, such as oocyte and embryo quality as well as pregnancy outcomes, most likely because they are transient.

A previous study illustrated that DBP increases as estrogen increases, for example during pregnancy and under hormone replacement therapies (6). This increase in DBP is associated with an increase in total 25(OH)D and a decrease in free 25(OH)D (33, 34), which explains our results that the DBP level is positively related to total 25(OH)D and negatively related with free 25(OH)D during ovarian stimulation, through E2 elevation. In **Table 1** of our study, age is slightly different among DBP groups. We further made linear regression analysis and it turns out that age is positively related to vitamin D binding protein (Adjusted β:0.043, 95% CI: 0.02–0.039, P=0.029). However, the IVF/ICSI outcomes are not significant even if we adjusted all the confounders.

The association of E2 with DBP increase was highly significant, but overall, the effect of E2 was moderate, this indicates that besides E2, a lot of other factors contribute to the regulation of hepatic DBP synthesis after hyperstimulation. As of today, there are a limited number of studies analyzing DBP and infertility and/or the impact of DBP on assisted reproductive technology (ART) outcomes. Franasiak et al. analyzed DBP and 25(OH)D forms in infertile women and showed that the DBP level was lower in infertile patients compared to fertile controls (35). A small study including 20 women suggested that DBP level in follicular fluid was reduced in women who didn’t become pregnant after ART, and those from whom fewer oocytes and fertilized eggs were obtained (36). However, considering the small number of participants in all the above-mentioned studies in ART populations, findings need independent confirmation in larger studies. Besides this study, there is no published report focusing on the serum concentration of DBP and its impact on IVF outcomes in animals nor humans. Thus, we analyzed DBP and IVF-related outcomes for the first time in an adequately powered study and found a strong correlation between the increase in E2 after stimulation for oocyte retrieval and DBP at the time of embryo transfer as well as a positive correlation between DBP and total 25(OH)D but a negative correlation between DBP and free 25(OH)D. However, this – most likely transient – decrease in free vitamin D had no impact on any embryo or pregnancy outcomes such as biochemical pregnancy rate and live birth rate in our relatively large study. This fits very well with our very recent study showing that neither free nor total vitamin D affected the risk of gestational diabetes in the same cohort (37). We assume that the observed changes in the proportion of adequate levels of free vitamin D in our study are transient because, after the E2-induced increased synthesis of DBP and a presumably stable further supply with 25(OH)D during ovarian stimulation, newly synthesized DBP binds free 25(OH)D and hence decrease its concentration further. This is a transient effect because when E2 levels reach a stable level with a further stable 25(OH)D supply, a new equilibrium will be established (see formula describing the relationship between free25(OH)D and DBP in the method section). This concept fits the observation that we did not see any effect on embryo and pregnancy outcomes.

On the other hand, it is well known that structural alterations of DBP are linked to alterations in clinical outcomes. There are several alleles of DBP such as Gc1f, Gc1s (rs7041 locus), and Gc2 (rs4588 locus), which differ in their binding affinity to vitamin D metabolites and have been variably associated with several clinical conditions (4). Schwartz et al. reported that different DBP haplotypes are associated with variations of total 25(OH)D, free 25(OH)D, and DBP levels. The lowest total and free levels of 25(OH)D were seen in the Gc 2/2 haplotype, which also tends to have the lowest DBP levels (38). Other studies have also found lower total 25(OH)D levels in subjects with the Gc2 allele (39, 40). Wang’s study illustrated that Gc rs16847024 and Gc rs3733359 were associated with an increased risk for gestational diabetes, compared to other DBP variants (41). In the CHARGE study with 1581 children and their parents, the DBP rs4588 variant was associated with the development of autism spectrum disorder (42).

We used a formula to calculate vitamin D binding protein instead of direct ELISA methods to determine vitamin D binding protein concentrations in our study. The biological property and the concentration of vitamin D binding protein are described by a formula (see method section). This formula has been used in many well-recognized studies from independent groups worldwide to calculate free vitamin D (12, 16, 43–48). Thus, this formula is also a valid tool to calculate vitamin D binding protein. The quality of the results depends on the quality of the methods employed to determine free and total vitamin D as well as albumin. We used in our study methods that have been certified for clinical use. The calculation method for vitamin D binding property is based on its biological activity (binding vitamin D which is expressed by the formula). This might overcome some disadvantages of using direct ELISAs to measure vitamin D protein concentrations such as Cross-reactivity: ELISA kits may exhibit cross-reactivity with other proteins or molecules that share structural similarities with DBP. This can lead to inaccurate results if not properly controlled (49).

Limited specificity: ELISA assays may not always be highly specific for DBP, as they can sometimes detect other proteins with similar epitopes. This can lead to false-positive or false-negative results (50).

Variability in antibodies: The quality and specificity of antibodies used in ELISA can vary between batches and suppliers. This can introduce variability in the results and might make it challenging to compare data from different studies (51). Limited dynamic range: ELISA assays may have a limited dynamic range, which means they may not accurately measure DBP concentrations across a wide range of concentrations. Dilution or concentration of samples may be necessary, which can introduce errors (52). Taken together direct measurement of DBP has also disadvantages and calculating vitamin D binding protein concentrations is a valid method to determine vitamin D binding protein concentrations if suitable methods are used for the analysis of free and total vitamin D as well as albumin. Head-to-head comparisons of both methods would be of interest.

Although our sample size is relatively large, making our findings robust, there are still some limitations in our study. Firstly, it is a single-center study, which means that we cannot fully exclude center-related confounding. Next, we did not consider genetic variants of DBP in our population. Finally, DBP levels in our study were calculated instead of measured directly, see above. As discussed the equations describing the relationship between free vitamin D, total vitamin D, albumin, and DBP, have been well-established for decades and are suitable to calculate the concentrations of components of the equation (53, 54).

In the current study, we demonstrated that DBP concentration correlates very well with the degree of E2 increase after ovarian stimulation. DBP is also positively correlated with total 25(OH)D and inversely with free 25(OH)D. Assuming a constant oral intake of vitamin D during ovarian hyperstimulation the fraction of free vitamin D must decrease after hyperstimulation due to the increase of vitamin D binding protein. An inverse relationship – in contrast to total vitamin D, see above - is clearly to be expected and was indeed seen in our study resulting in a – most likely transient - decrease in the proportion of free 25(OH)D during ovarian stimulation caused by E2-stimulated DBP synthesis. However, such alterations do not affect clinical IVF/ICSI outcomes.

## Data availability statement

The raw data supporting the conclusions of this article will be made available by the authors, without undue reservation.

## Ethics statement

The studies involving humans were approved by the Ethics Committee of the Reproductive and Genetic Hospital of CITIC-Xiangya. The studies were conducted in accordance with the local legislation and institutional requirements. The participants provided their written informed consent to participate in this study.

## Author contributions

HC: Data curation, Formal analysis, Funding acquisition, Investigation, Methodology, Project administration, Software, Validation, Visualization, Writing – original draft, Writing – review & editing. JY: Data curation, Formal analysis, Investigation, Methodology, Project administration, Software, Writing – original draft, Writing – review & editing. LH: Supervision, Writing – review & editing. YL: Writing – review & editing. J-GH: Writing – review & editing. XZ: Software, Writing – review & editing. AH: Software, Writing – review & editing. GL: Supervision, Writing – review & editing. FG: Supervision, Writing – review & editing. BH: Conceptualization, Supervision, Validation, Writing – original draft, Writing – review & editing.
